# Untangling the complex relationships between incident gout risk, serum urate, and its comorbidities

**DOI:** 10.1186/s13075-018-1558-3

**Published:** 2018-05-03

**Authors:** Mengying Sun, Ana I. Vazquez, Richard J. Reynolds, Jasvinder A. Singh, Mathew Reeves, Tony R. Merriman, Angelo L. Gaffo, Gustavo de los Campos

**Affiliations:** 1 Department of Epidemiology and Biostatistics, Michigan State University, 220 Trowbridge Rd, East Lansing, MI 48824 USA; 2 The Institute for Quantitative Health Science and Engineering, Michigan State University, 220 Trowbridge Rd, East Lansing, MI 48824 USA; 3 Division of Clinical Immunology and Rheumatology, University of Alabama Birmingham (UAB), 1825 University Blvd., Birmingham, AL 35294 USA; 4 Biochemistry Department, School of Biomedical Sciences, University of Otago, 710 Cumberland St., Dunedin, 9054 New Zealand; 5 Department of Probability and Statistics, Michigan State University, 220 Trowbridge Rd, East Lansing, MI 48824 USA

**Keywords:** Gout, Serum urate, Comorbidities, ARIC, Ethnic differences, Obesity

## Abstract

**Background:**

Many gout comorbidities (e.g., hypertension) are correlated with serum urate. In this investigation, we identified risk factors (e.g., systolic blood pressure [SBP]), that (1) are associated with incident gout, (2) have effects on gout risk that cannot be fully explained by correlated differences in serum urate, and (3) may modulate the relationship between gout and serum urate.

**Methods:**

Using data from the Atherosclerosis Risk in Communities (ARIC) study, we estimated the unadjusted associations between gout and risk factors by calculating ORs and using chi-square tests. The adjusted associations were analyzed using logistic regression by sequentially adding (1) one risk factor at a time or (2) all risk factors, to a baseline model that includes serum urate only. Stepwise selection was used to select main effects. Two-way interactions of variables from the main effects model were also analyzed.

**Results:**

Average gout incidence was 2.7 per 1000 people per year. Serum urate was highly associated with incident gout, with odd ratios of 3.16 [95% CI 2.11, 4.76] and 25.9 [95% CI 17.2, 38.4] for moderately high (6–8 mg/dl) and high serum urate (> 8 mg/dl), relative to normal serum urate (< 6 mg/dl), respectively. Ethnicity and SBP were independently and additively associated with gout after accounting for serum urate levels. No significant interactions were found between serum urate and ethnicity or SBP.

**Conclusions:**

Ethnicity and hypertension are predictive of gout risk, and the associations cannot be fully explained by serum urate. For serum urate levels near the crystallization threshold (6–8 mg/dl) African Americans and people with hypertension are at two to three times greater risk for developing gout. The gout risk for this group appears to increase before the onset of severe hyperuricemia.

**Electronic supplementary material:**

The online version of this article (10.1186/s13075-018-1558-3) contains supplementary material, which is available to authorized users.

## Background

Hyperuricemia (serum urate concentration > 7 mg/dl) is the most important risk factor for gout. At this physiological threshold and above, monosodium urate crystals, which are necessary to initiate gout flares, form and the incidence of gout increases substantially [1]. However, even lower serum urate levels (> 6 mg/dl) can also lead to formation of monosodium urate crystals at high temperature [2]. Hyperuricemia and gout are strongly associated with comorbidities such as obesity, hypertension, type 2 diabetes, renal disease, and cardiovascular disease. Numerous epidemiological studies have demonstrated that the prevalence of these comorbidities is higher in patients with gout. For example, among individuals with gout, 63% have metabolic syndrome compared with 25% in people without gout [3]. Similar observations are noted for cardiovascular disease, chronic kidney disease and obesity [4–7].

Some of these comorbidities are also associated with gout development. Results from the Atherosclerosis Risk in Communities (ARIC) study suggest that the incidence of gout among people with hypertension is nearly three times that in normotensive people (4.6% versus 1.5%) [8]. Likewise, African Americans are at increased risk of incident gout [9]. In those studies hypertension and ethnicity were each independently associated with gout when accounting for levels of serum urate. This result is interesting because serum urate is associated with incident hypertension [10], and hypertension has been demonstrated to be higher in African Americans [11]. If serum urate is related to hypertension and hypertension to gout, it might be expected that accounting for serum urate levels in a multivariable model would nullify the association of hypertension with gout. Evidently, hypertension and ethnicity as well as perhaps other variables are important to predicting the development of gout over and beyond the information provided by serum urate alone. However, there are no studies that have explicitly considered the additive and interactive combined effects as important explanatory variables for gout development in the follow-up period. Results from such models could provide important information for clinicians in describing the likely future course of disease to patients at risk for gout.

More research is needed into whether these factors have a direct causal effect on the risk of developing gout or if the association between these risk factors and gout is mediated by hyperuricemia. In this paper, we present results of a study adding novel information to the literature on how important risk factors for incident gout (e.g., hypertension) are mediated by serum urate. We also consider whether features in the model are likely to be directly involved with gout pathogenesis by comparing and contrasting which variables are associated with incident and prevalent gout, respectively. Our specific aims were to (1) quantify the effects of serum urate, as a predictor, on the risk of incident gout; (2) determine whether sex, ethnicity, body mass index (BMI), and additional traits representing gout comorbidities are associated with the risk of incident gout after accounting for differences in serum urate; and (3) determine whether the effect of serum urate on incident gout differs depending on sex, ethnicity, and comorbid conditions.

## Methods

### Materials

Data were obtained from the Atherosclerosis Risk in Communities (ARIC) [12] study. ARIC is a prospective epidemiologic study conducted in four U.S. communities during 1987–1998. The study collected information at baseline (1987–1988) and at three follow-up visits that were conducted every 3 years. Information on past history of gout was self-reported and obtained at the third follow-up visit (examination 4) by asking the question, “Has a doctor ever told you you had gout?” The age of gout onset was derived from the subsequent question, “How old were you when you were first told had gout?” Self-report of physician-diagnosed gout has good reliability and sensitivity and has been used in previous epidemiologic studies [13–16]. Incident gout was defined as gout that occurred during the study (i.e., between examinations 1 and 4). Prevalent gout at examination 1 was defined as gout with onset prior to age at baseline.

Clinical variables were assessed at the first baseline visit and included demographics (sex, ethnicity, and age) and serum urate (mg/dl); BMI (kg/m2), systolic blood pressure (SBP; mmHg), glucose (mmol/L), high-density lipoprotein (HDL; mmol/L), low-density lipoprotein (LDL; mmol/l), creatinine (mg/dl), and triglycerides (mmol/L) were chosen for their representation of important comorbidities of gout: obesity, hypertension, type 2 diabetes, renal disease, and cardiovascular disease. The estimated glomerular filtration rate (eGFR) was derived using the Modification of Diet in Renal Disease (MDRD) equation [17, 18]. For ease of interpretation, these clinical variables were dichotomized into normal or high-risk levels using commonly accepted thresholds as described in Table 1. Given the importance of serum urate, it was included as a quantitative (continuous variable) or categorical risk factor with three levels.

**Table 1:** Thresholds used to define risk levels

Variable	Unit	Levels
Serum urate	mg/dl	Normal: < 6; medium-high, 6–8; high: > 8
Body mass index	kg/ m^2^	Non-obese: ≤ 30; obese: > 30
Systolic blood pressure	mm Hg	Normal or prehypertensive: ≤ 140; hypertensive: > 140
Glucose	mmol/l	Normal: ≤ 7; high: > 7
HDL cholesterol	mmol/l	Low: < 1; normal: ≥ 1
LDL cholesterol	mmol/l	Normal: ≤ 3.4; high: > 3.4
eGFR	mL/min/1.73 m^2^	Low: < 60; normal or mildly reduced: ≥ 60
Triglycerides	mmol/l	Normal: ≤ 1.7; high: > 1.7

The thresholds used to define normal and high levels, and unit conversion ratios were obtained from other sources [19–23]

### Statistical analysis

Our primary objective was to identify factors that affect the risk of developing gout; therefore, the focus of our analyses was primarily on incident gout. However, we also carried out analysis for prevalent gout to understand which factors are associated with existing gout at baseline.

For both prevalent and incident gout, we first assessed the unadjusted (marginal) association of each risk factor and gout separately using ORs and chi-square tests. Subsequently, we fit a series of statistical models to address each aim of our study. Because gout was assessed at examination 4 and we considered only individuals with gout data, we did not have loss to follow-up; therefore, our primary analytic method was logistic regression (gout yes/no). Our baseline model consisted of a logistic regression of gout on serum urate (continuous) only; we also considered using splines to accommodate departures from linearity. We then assessed the effects of other risk factors after accounting for differences in serum urate. For this purpose, we used three approaches: (1) adding to the baseline model one risk factor at a time, (2) fitting a model including all the risk factors considered in the study, and (3) forward selection using a stepwise procedure in which serum urate was forced to enter the model. The stepwise regression was implemented using the Akaike information criteria (AIC) and Bayesian information criterion (BIC) [24, 25]. The BIC penalizes model complexity more strongly than AIC and therefore tends to favor more parsimonious models. Finally, we tested for the existence of interactions between serum urate and risk factors using the additive model selected from the previous stage. Taking this as the baseline main effect model, we tested for interactions between the factors entering that model and serum urate using three approaches: (1) adding interactions one factor at a time, (2) adding all two-way interactions simultaneously, and (3) using a stepwise procedure for selection of interactions.

All analyses were conducted in the R environment [26]. Logistic regressions were implemented using the glm function, and stepwise search was conducted using the step function, both of which are available in the base package of R. Natural splines were implemented using the ns function in the splines package. Odds ratios were calculated using the oddsratio function in fmsb package of R [27].

## Results

Among a starting population of 9503, we excluded 7.2% leaving 8818 individuals who met our study entry criteria. Of these, 56.2% (4959) were females; 80.2% (7071) were white, and 19.8% (1747) were African American. Reasons for excluding subjects included (1) no information on gout (*n*=119), (2) missing values for at least one risk factor (*n*=245), or (3) had gout before cohort entry (for incident gout only) (*n*=321). Descriptive statistics for all risk factors are shown in Additional file 1.

The age at the first visit ranged from 44 to 66 (53.92 ± 5.69) years. Table 2 shows the percentage of subjects and gout incidence for demographic and clinical factors. There were 216 gout cases among the 8818 subjects over the 9-year follow-up. The average number of new gout cases was 2.7 per 1000 people per year. For most risk factors, the association was moderate to strong, with an OR typically equal to or greater than 2. As expected, for serum urate, the association was even stronger, with an OR of 25.9 (95% CI 17.2, 38.4) for the high level compared with the normal level. Results for prevalent gout and risk factors are shown in Additional file 2. Ethnicity and glucose differed most between incident gout and prevalent gout with ORs of 2.7 (95% CI 2.01, 3.51) versus 1.0 (95% CI 0.77, 1.34) for ethnicity and 1.6 (95% CI 0.99, 2.42) versus 2.7 (95% CI 1.97, 2.63) for glucose, respectively.

**Table 2:** Unadjusted univariate association between incident gout and clinical covariates assessed at baseline

Risk factor	Percentage	Gout	OR	*p*-value	
	(%)	incidence^a^	(95% CI)	For OR	Chi-square test
Overall	100	2.72			
Serum urate				<.001	<.001
Normal	54.0	0.79	Ref		
Medium-high	38.2	2.47	3.16 (2.11, 4.76)		
High	7.7	17.43	25.9 (17.2, 38.4)		
Sex				<.001	<.001
Female	56.2	1.90	Ref		
Male	43.8	3.77	2.01 (1.53, 2.66)		
Ethnicity				<.001	<.001
European American	80.2	2.07	Ref		
African American	19.8	5.34	2.66 (2.01, 3.51)		
Age				0.325	0.36
≤ 54 years	54.7	2.56	Ref		
> 54 years	45.3	2.92	1.15 (0.87, 1.50)		
Body mass index				<.001	<.001
Nonobese	75.1	2.21	Ref		
Obese	24.9	4.25	1.96 (1.48, 2.59)		
eGFR				<.001	<.001
Low	2.1	8.41	3.42 (1.95, 6.00)		
Normal or mildly reduced	97.9	2.60	Ref		
HDL cholesterol				0.001	0.002
Low	21.3	3.85	1.62 (1.20, 2.17)		
Normal	78.7	2.42	Ref		
LDL cholesterol				0.613	0.662
Normal	46.5	2.82	Ref		
High	53.5	2.64	0.93 (0.71, 1.22)		
Systolic blood pressure				<.001	<.001
Normal or prehypertensive	89.0	2.42	Ref		
Hypertensive	11.0	5.17	2.19 (1.57, 3.06)		
Triglycerides				<.001	<.001
Normal	74.6	2.18	Ref		
High	25.4	4.32	2.02 (1.53, 2.67)		
Glucose				0.055	0.075
Normal	93.1	2.63	Ref		
High	6.9	4.00	1.55 (0.99, 2.42)		

^a^Average number of new cases per 1000 patients per year

### Baseline model

**Fig. 1:**
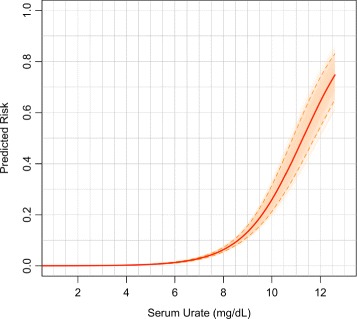
Predicted risk of developing gout by serum urate only. *Peach-colored lines* were obtained using 1000 bootstrap samples; the *red dashed lines* depict 95% confidence bands

Figure 1 shows the predicted risk of incident gout by serum urate from logistic regression. Note that although serum urate was entered linearly in the model, owing to the nonlinear mapping of the logistic link, the relationship between gout risk and serum urate was nonlinear. Indeed, our results show an exponential increase in risk for subjects with serum urate between 5 and 8.5 and an approximately linear increase in risk for individuals with serum urate levels between 9 and 12. The results obtained when serum urate was included in the model using a natural spline (Additional file 3) were very similar to those obtained with the linear specification; therefore, hereinafter we maintain serum urate entering linearly. Additional file 4 displays the estimated risk of prevalent gout by serum urate.

### Effects of other risk factors

We also tested for associations between gout and other risk factors after accounting for differences in levels of serum urate using the three approaches described in the “Methods” section above. Table 3 shows the results obtained for each risk factor and each approach considered. In the two-factor models, ethnicity and SBP showed significant influence on the risk of incident gout with *p* values less than 0.01. LDL cholesterol showed influence on gout with a *p* value 0.015. All other variables did not show significant effects. When considering all factors together, the results were similar to those derived from the two-factor models. In stepwise procedures using AIC, ethnicity and SBP entered in the model (*p* value < 0.01). With the BIC, only ethnicity was entered in the model with serum urate.

**Table 3:** Adjusted association analysis for incident gout by risk factor using three different approaches

	Two-factors		Full		Stepwise regression			
	regression^a^		regression^b^		(AIC)^c^		(BIC)^c^	
	Est. (OR)	*p* Value	Est. (OR)	*p* Value	Est. (OR)	*p* Value	Est. (OR)	*p* Value
Serum urate (mg/dl)	-	-	.792 (2.21)	<**.001**	.805 (2.24)	<**.001**	.795 (2.21)	<**.001**
Sex: Male	-.121 (0.89)	0.424	.030 (1.03)	0.854	-	-	-	-
Ethnicity: AA	.674 (1.96)	<**.001**	.647 (1.91)	<**.001**	.622 (1.86)	<**.001**	.675 (1.91)	<**.001**
Age, years	-.001 (1.00)	0.959	.000 (1.00)	0.974	-	-	-	-
Glucose: High	.080 (1.08)	0.750	-.055 (0.95)	0.830	-	-	-	-
HDL: Low	-.148 (0.86)	0.350	-.046 (0.95)	0.796	-	-	-	-
LDL: High	-.354 (0.70)	0.015	-.381 (0.68)	0.010	-.373 (0.69)	0.011	-	-
Triglycerides: High	-.057 (0.94)	0.710	.093 (1.10)	0.579	-	-	-	-
SBP: Hypertensive	.580 (1.79)	**0.002**	.510 (1.66)	**0.006**	.508 (1.66)	**0.005**	-	-
BMI: Obese	.054 (1.06)	0.723	-.044 (0.96)	0.786	-	-	-	-
eGFR: Low	.364 (1.44)	0.246	.042 (1.04)	0.200	-	-	-	-

^a^Logistic regression of gout on two predictors: serum urate plus one of the factors in rows

^b^Logistic regression of gout all the factors listed in rows.

^c^Stepwise logistic regression. Rows with no results correspond to predictors that did not entered in the final model. *p* Values correspond to estimated coefficients. Bold indicates effect estimates that were statistically different from zero at 0.01 significance level

Similarly, Additional file 5 shows the corresponding results for prevalent gout. Sex, age, glucose and BMI were statistically significant in all three approaches.

**Fig. 2:**
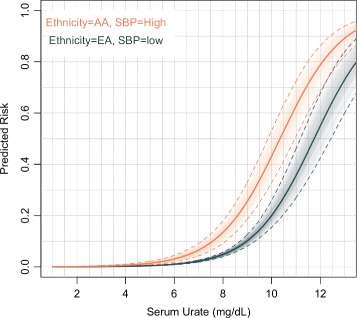
Estimated risk of incident gout versus serum urate by risk groups. *AA* African American, *EA* White, *SBP* Systolic blood pressure. Thin lines correspond to estimates derived using 1000 bootstrap samples; the *dashed lines* give 95% confidence bands

Figure 2 shows the predicted risk of incident gout by serum urate, controlling for the effects of ethnicity and SBP. These predictors were derived from the model selected with stepwise procedure and showed significance at 0.01 significance level. For any given level of serum urate, African Americans with high SBP had higher risk of developing gout than European Americans with normal SBP. The OR between high- and low-risk groups for people with serum urate concentrations between 6 and 8 mg/dl was 3.03. The bootstrap confidence bands displayed in Fig. 2 indicate that the differences in risk between European Americans with normal SBP and African Americans with high SBP is statistically different from zero. Analysis of prevalent gout again showed a different result, but with similar pattern, on gender and glucose (Additional file 6).

### Interaction analysis

After examining the main effects of various risk factors, we assessed whether the effect of serum urate on gout risk was modulated by other risk factors. The additive baseline model for interaction analyses included the predictors that were selected by AIC in the stepwise regression and had significant effect at 0.01 level in the final model (Table 3). For incident gout, these were serum urate, ethnicity, and SBP. In our interaction analyses, we did not find any significant interactions between ethnicity and SBP with serum urate (Table 4). For prevalent gout, only sex was found to be significant in the interaction model (Additional file 7).

**Table 4:** Interaction analysis for incident gout by risk factor using three different approaches

	One interaction^a^		Full regression^b^		Stepwise^c^	
	Est. (OR)	*p* Value	Est. (OR)	*p* Value	Est. (OR)	*p* Value
Main effects						
Serum urate, (mg/dl)	-	-	0.846 (2.33)	<**0.001**	0.794 (2.21)	<**0.001**
Ethnicity: AA	-	-	1.622 (5.06)	0.025	0.624 (1.87)	<**0.001**
SBP: Hypertensive	-	-	0.529 (1.70)	0.579	0.486 (1.63)	**0.008**
Interactions						
SU: Ethnicity AA	-0.130 (0.88)	0.156	-0.130 (0.88)	0.16	-	-
SU: SBP Hypertensive	-0.026 (0.97)	0.833	-0.007 (0.99)	0.955	-	-

^a^Derived from a logistic regression of gout on main effects plus one interaction term. The main effect estimates were not printed because they depended on which interaction was included

^b^Derived from a logistic regression of gout on all main effects and the two interactions listed in rows

^c^Derived from a stepwise logistic regression that minimized either AIC or BIC Rows with ‘-’ correspond to predictors that were not selected by the stepwise procedure Bold cells indicate effect estimates that were statistically different than zero at 0.01 significance level

## Discussion

In this study, we sought to disentangle whether clinical variables representing comorbid conditions of gout were independent of or mediated by the prominent risk factor for gout, serum urate. We used marginal, two-factor, and stepwise regression with and without interactions to evaluate the evidence that clinical variables representing gout comorbidities were associated with incident gout. We have shown that SBP and ethnicity are independent risk factors for incident gout conditional on serum urate. The results demonstrate that the effects of ethnicity and hypertension are not completely mediated by serum urate.

These findings confirm and extend results from previous studies of incident gout with the ARIC cohort. A report by McAdams-DeMarco [8] showed that hypertension at baseline is associated with incident gout. Maynard et al. [9] demonstrated that African Americans have increased risk of incident gout compared with European Americans. A novel aspect of our analysis and results is that our best stepwise model included serum urate and both ethnicity and hypertension but not their interaction. Thus, we suggest that the effects of being African American and having high blood pressure are additive to the risk of developing gout in the future. The risk of gout increases exponentially as a function of serum urate and is always higher for hypertensive African Americans than for normotensive European Americans. For example, given a serum urate of 6 mg/dl at baseline, the risk of developing gout for hypertensive African Americans more than doubles to 3% from 1% for normotensive Europian Americans. At 8 mg/dl, the risk increases to 13% from 5% for hypertensive African Americans and European Americans, respectively. There is nonlinearity in the increase in gout risk especially in the 6–8 mg/dl range, however, even in an absence of interactions, owing to the nonlinear nature of the logistic link, an additive model produces interaction-like patterns.

With strong support for the additive effect of ethnicity and hypertension on gout risk, we ask: What are potential confounders and what are the possible implications for pathogenesis? In the ARIC cohort, white individuals were, on average, 1 year older at baseline than African Americans (Additional file 8). Thus, one could argue that in part the effect of ethnicity may be confounded by age; however, when all predictors were fitted jointly, age did not have significant effect on gout risk, nor was there an age-by-ethnicity interaction effect on incident gout. Similarly, baseline mean SBP and serum urate were higher in African Americans than in European Americans. The percentage of participants taking hypertension-lowering medication within the past two weeks prior to visit 1 was 41.6% for African American versus 22.8% for white individuals. One could argue that diuretic use by African Americans may cause higher gout risk because it is known that these antihypertensive medications also increase serum urate level [28]. However, if this were the only explanation for the increased gout risk, given the fact that previous studies of diuretic use and increased gout risk show that the effect is nullified by accounting for serum urate level [8], this hypothesis cannot explain why ethnicity has an effect on incident gout even after conditioning on serum urate levels. Another possibility is that an unobserved variable is associated with hypertensive African Americans. For example, poor kidney function, perhaps as a result of hypertension, could increase gout risk in African Americans. However, the absence of an eGFR and ethnicity interaction effect do not support this interpretation, although we may not have a sufficient number of events to definitively study this potential confounder.

There is a large body of literature showing that serum urate is causal on hypertension and hypertension prevalence is higher in African Americans [29, 30]. Hyperuricemia causing hypertension is also sustained by animal models [31]. Our results demonstrate that serum urate does not completely mediate this pathway to gout and that both hypertension and ethnicity independently and additively increase the risk of incident gout. Furthermore, for hypertensive African Americans, there seems to be an increased risk at relatively moderate serum urate levels (< 8 mg/dl). Therefore, the events that lead to gout among African Americans and those experiencing hypertension may have their beginnings when serum urate concentration is at seemingly benign levels. Finally, hypertension may represent a biomarker of poor renal function and a number of additional comorbidities that are associated with increased serum urate and ultimately gout risk. The pathological mechanism potentially influencing effects of serum urate on endothelial function, renal dysfunction, and blood pressure at these levels are addressed elsewhere [8, 32].

Our conditional association analysis indicated the presence of three groups of incident gout predictors. The first group is discussed in detail above, including ethnicity and SBP. A second group of variables includes sex, BMI, eGFR, HDL cholesterol, and triglycerides; these factors had a statistically significant marginal association with incident gout; however, the association became nonsignificant once serum urate level was accounted for. This suggests that the effects of these risk factors can be fully explained (or can be considered as mediated) by serumurate levels. The third includes age, LDL, and glucose, which showed no marginal association with incident gout. It is note worthy that glucose, age, and BMI were not associated with incident gout but were associated with prevalent gout. BMI at baseline has been associated with a 10-year increase in serum urate, which is consistent with the result that serum urate mediates this relationship with incident gout [33, 34]. These differences address an important issue of how to interpret association of gout and its comorbidities through two different outcomes: incident and prevalent gout. Another novel aspect of our results is that they suggest that although BMI and glucose are unconditional risk factors for incident gout, the differences in obesity and type 2 diabetes rates observed in people with gout [4] may increase subsequent to first gout flare. This highlights the comorbid burden that accompanies gouty arthritis and contributes to low health-related quality of life indicators for people with gout [35, 36].

## Conclusion

Our analysis confirms that serum urate is a strong predictor of incident gout. Many demographic and clinical covariates that have marginal associations with incident gout (sex, glucose, BMI, eGFR) do not longer show association with that outcome after differences in serum urate are accounted for. However, ethnicity and hypertension have an association with incident gout that is not fully explained by interindividual differences in serum urate. For serum urate levels near the crystallization threshold (6–8 mg/dl) African Americans and hypertensive people are at 2–3 times the risk for developing gout. The disparity in obesity and type-2 diabetes may increase once gout is established, which needs further investigation inlarger prospective cohort studies.

## Additional files

Additional file 1 **Table S5**. Summary statistics for quantitative variables. (JPG 102 kb)

Additional file 2 **Table S6**. Unadjusted univariate association between prevalent gout and clinical covariates assessed at baseline. (JPG 127 kb)

Additional file 3 **Figure S3**. Estimated risk of incident gout by serum urate only. Serum urate was entered either linearly or nonlinearly. (PNG 68 kb)

Additional file 4 **Figure S4**. Estimated risk of prevalent gout by serum urate only. (PNG 142 kb)

Additional file 5 **Table S7**. Adjusted association analysis for prevalent gout by risk factor using three different approaches. (JPG 165 kb)

Additional file 6 **Figure S5**. Estimated risk of prevalent gout versus serum urate by risk groups. (PNG 263 kb)

Additional file 7 **Table S8**. Interaction analysis for prevalent gout by risk factor using three different approaches. (JPG 236 kb)

Additional file 8 **Table S9**. Mean value of each risk factor by ethnicity group. (JPG 100 kb)

## Abbreviations

AA: African americans
BMI: Body mass index
EA: European Americans
SBP: Systolic blood pressure
