# Supplementary figures and images for: Untangling the complex relationships between incident gout risk, serum urate, and its comorbidities
Source: Arthritis Res Ther. 2018 May 3;20:90. doi: 10.1186/s13075-018-1558-3 (PMC5932762; doi:10.1186/s13075-018-1558-3)

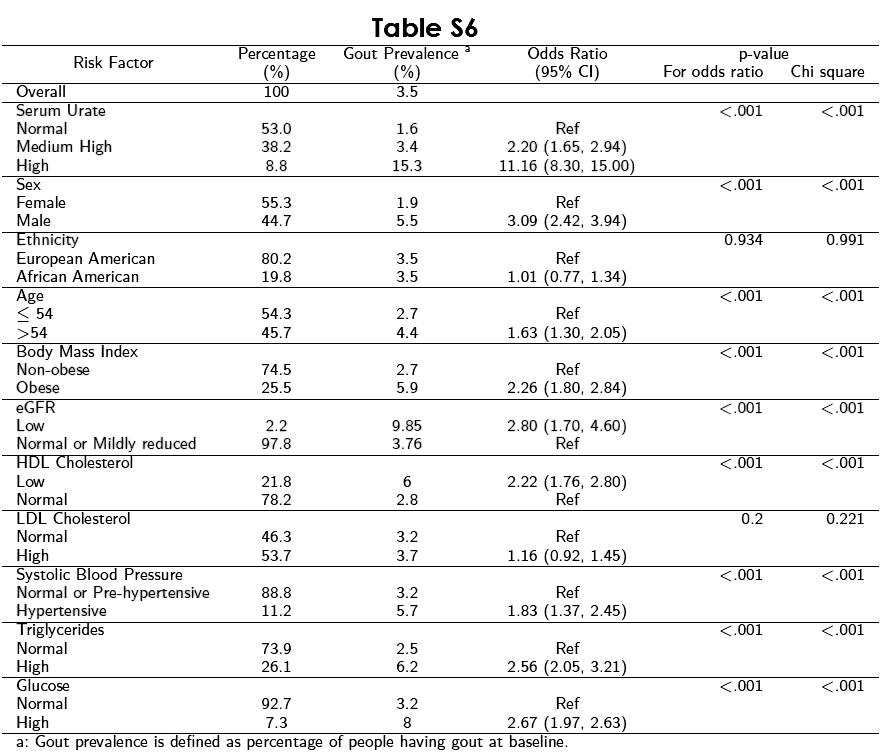

Supplement: Supplementary file 1 — Table S5. Summary statistics for quantitative variables. (JPG 102 kb) [file 13075_2018_1558_MOESM1_ESM.jpg]

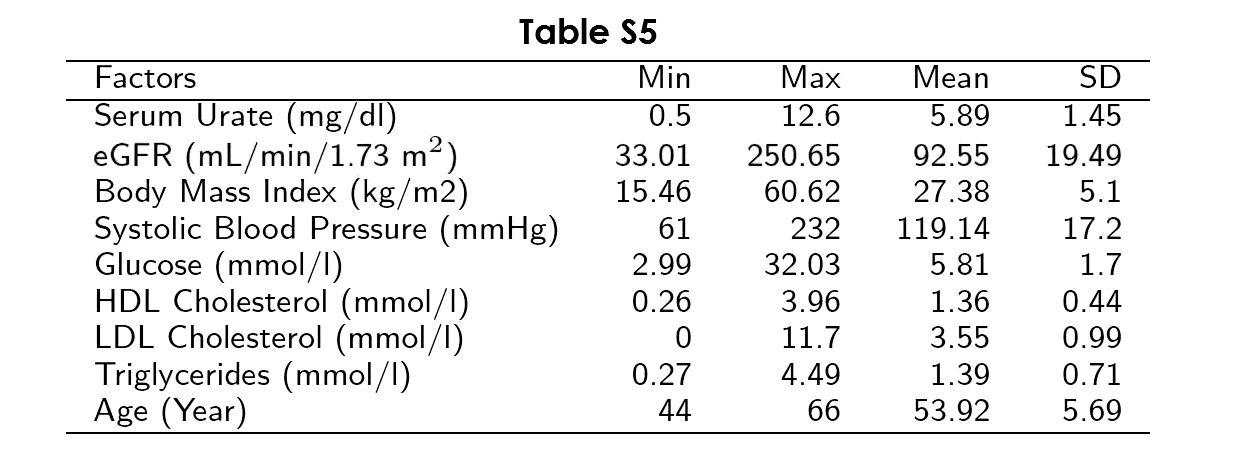

Supplement: Supplementary file 2 — Table S6. Unadjusted univariate association between prevalent gout and clinical covariates assessed at baseline. (JPG 127 kb) [file 13075_2018_1558_MOESM2_ESM.jpg]

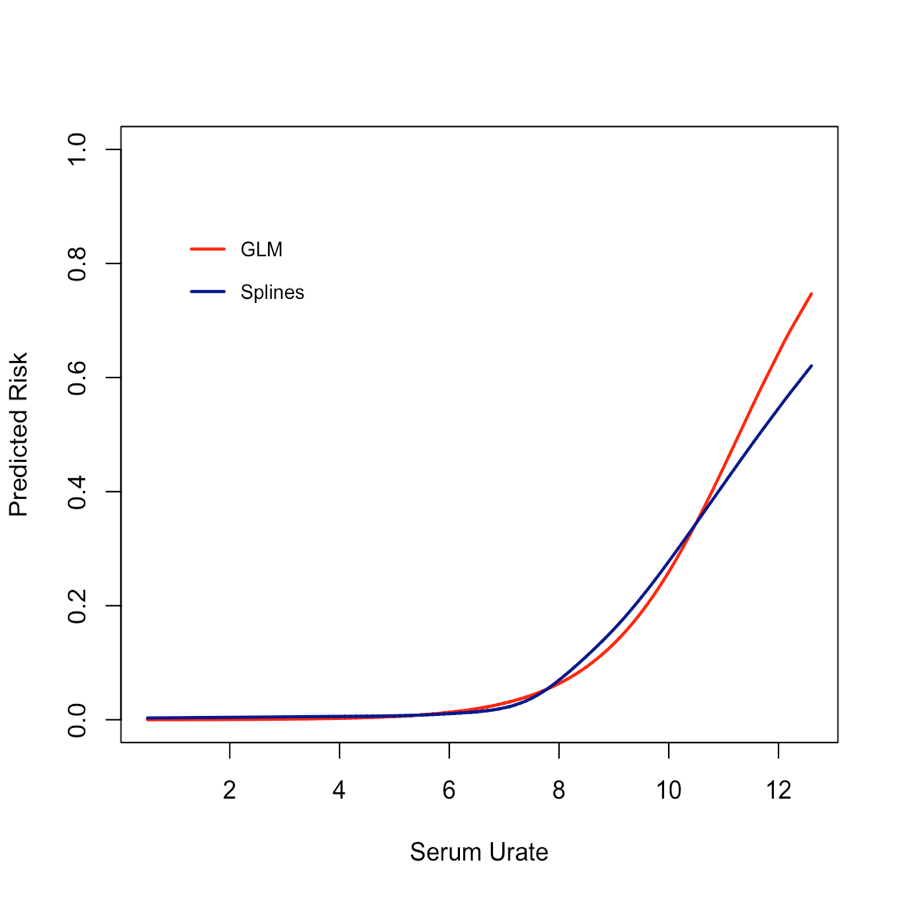

Supplement: Supplementary file 3 — Figure S3. Estimated risk of incident gout by serum urate only. Serum urate was entered either linearly or nonlinearly. (PNG 68 kb) [file 13075_2018_1558_MOESM3_ESM.png]

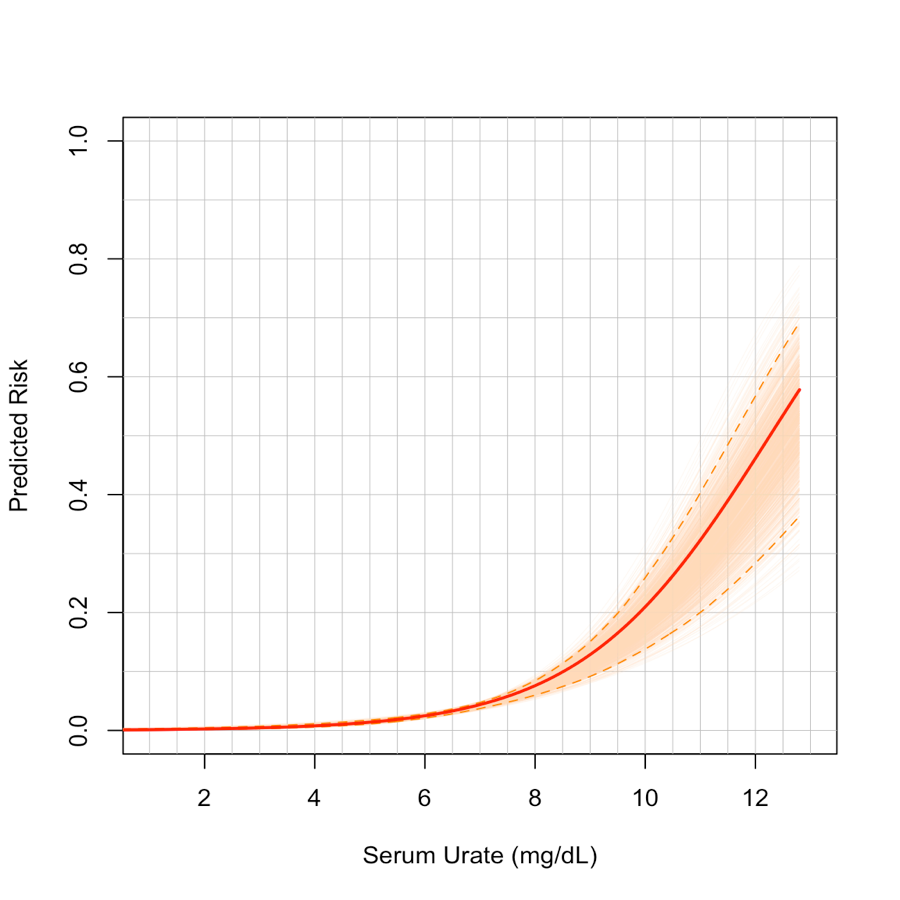

Supplement: Supplementary file 4 — Figure S4. Estimated risk of prevalent gout by serum urate only. (PNG 142 kb) [file 13075_2018_1558_MOESM4_ESM.png]

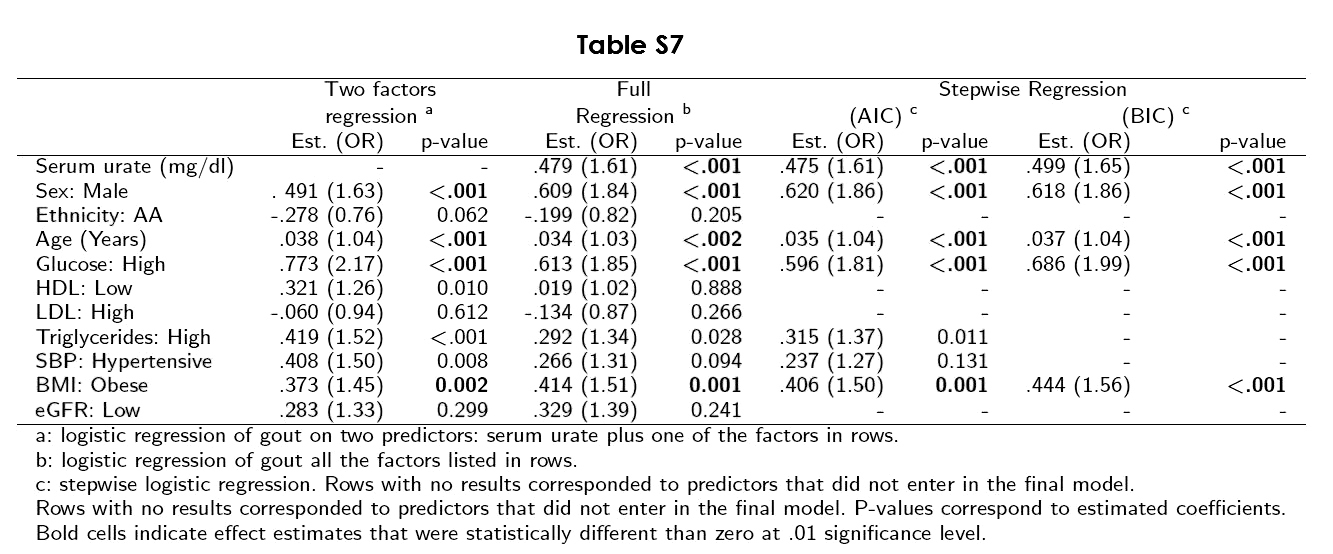

Supplement: Supplementary file 5 — Table S7. Adjusted association analysis for prevalent gout by risk factor using three different approaches. (JPG 165 kb) [file 13075_2018_1558_MOESM5_ESM.jpg]

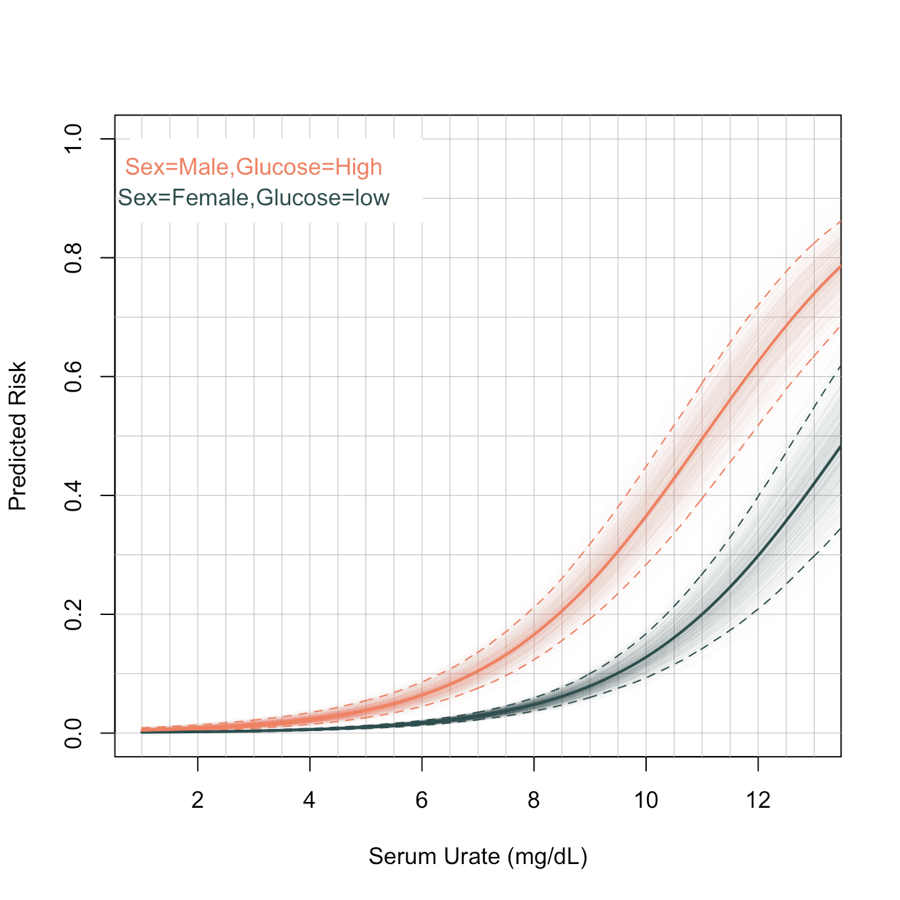

Supplement: Supplementary file 6 — Figure S5. Estimated risk of prevalent gout versus serum urate by risk groups. (PNG 263 kb) [file 13075_2018_1558_MOESM6_ESM.png]

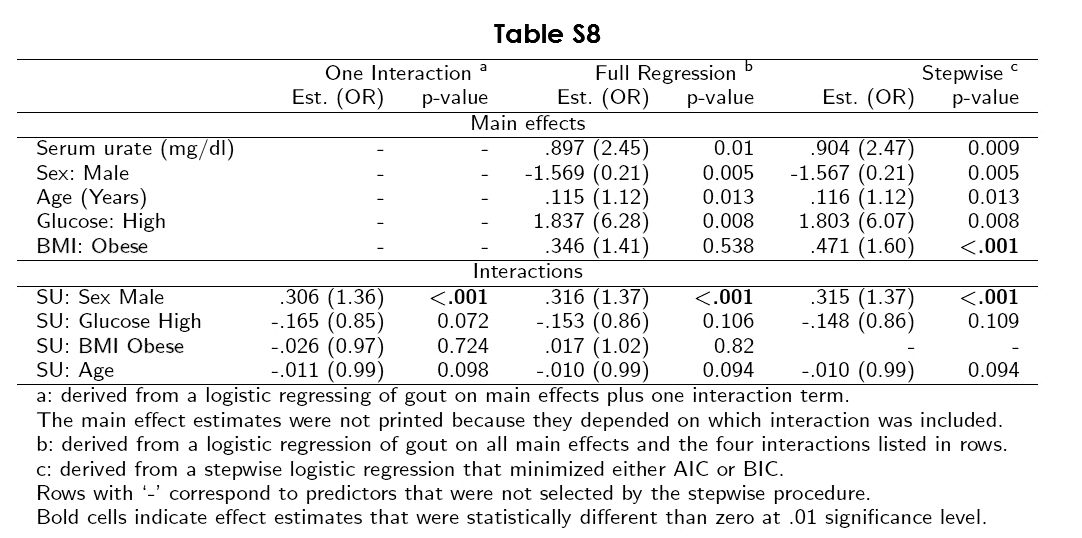

Supplement: Supplementary file 7 — Table S8. Interaction analysis for prevalent gout by risk factor using three different approaches. (JPG 236 kb) [file 13075_2018_1558_MOESM7_ESM.jpg]

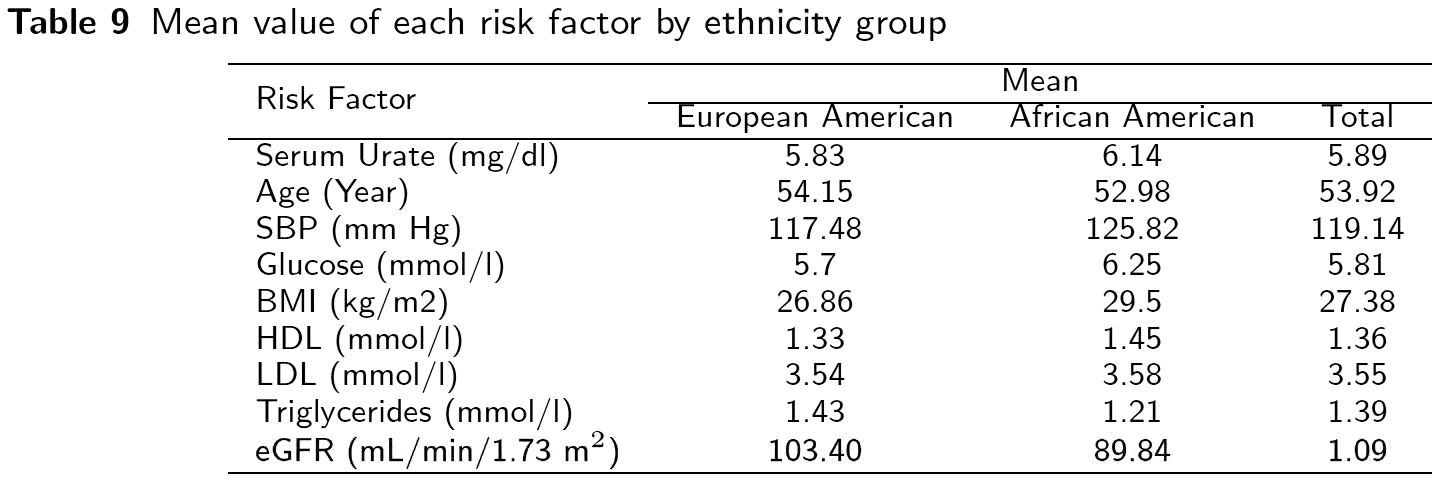

Supplement: Supplementary file 8 — Table S9. Mean value of each risk factor by ethnicity group. (JPG 100 kb) [file 13075_2018_1558_MOESM8_ESM.jpg]
